# Upper-Limb asymmetry in wheelchair para-athletes: a comparative analysis across sports disciplines

**DOI:** 10.3389/fspor.2026.1858941

**Published:** 2026-07-08

**Authors:** Marco Kokaly, Antonia Carvallo, Catalina Guzmán, María Jesús Prieto, Matías Henríquez, Oscar Valencia

**Affiliations:** 1 Escuela de Kinesiología, Universidad de los Andes, Santiago, Chile; 2 Escuela de Kinesiología, Facultad de Salud, Universidad Santo Tomas, Santiago, Chile; 3 Laboratorio Integrativo de Biomecánica y Fisiología del Esfuerzo, Escuela de Kinesiología, Universidad de los Andes, Santiago, Chile

**Keywords:** muscle strength, performance, range of motion, upper-limb asymmetry, wheelchair para-athletes

## Abstract

**Background:**

Wheelchair use has increased globally, with many individuals relying on wheelchairs not only for mobility but also for participation in sport. However, upper-limb (UL) asymmetries are common among wheelchair athletes and may contribute to overuse injuries. Limited evidence exists regarding how asymmetry differs among athletes in para-sports who use wheelchairs to compete. This study aimed to compare UL asymmetry levels among wheelchair para-athletes across different sports disciplines through an exploratory analysis.

**Methods:**

A cross-sectional study was conducted with 30 wheelchair para-athletes (27 men, 3 women; 18–40 years) competing in six disciplines: powerlifting, wheelchair rugby, table tennis, wheelchair basketball, boccia, and wheelchair handball. Muscle strength and range of motion (ROM) were assessed using a validated portable dynamometer/inclinometer across seven UL movements. Asymmetry was calculated as the percentage difference between dominant and non-dominant limbs, with values ≥15% classified as asymmetric.

**Results:**

Wheelchair handball (10%), basketball (12%), and powerlifting (10%) demonstrated predominantly symmetric profiles, whereas table tennis (17%), boccia (30%), and wheelchair rugby (26%) exhibited greater asymmetry. Significant differences between symmetric and asymmetric groups were observed in strength (shoulder abduction, flexion, extension, and internal rotation; *p* < 0.05) and ROM (shoulder abduction, adduction, and flexion; *p* < 0.05), with higher values recorded in the asymmetric group.

**Conclusion:**

UL asymmetry indices ranged from 10% to 30% across disciplines, with table tennis, boccia, and rugby surpassing the 15% threshold. These differences in shoulder strength and ROM suggest that unilateral technical demands and impairment level are key determinants of musculoskeletal adaptation in wheelchair sport.

## 1 Introduction

According to the World Health Organization, the global population of wheelchair users has grown significantly, reaching approximately 80 million people (1). Wheelchairs play a crucial role in supporting independence, expanding opportunities, and enhancing the quality of life for individuals with disabilities. However, wheelchair propulsion has been characterized as an energetically and mechanically inefficient form of movement, requiring the activation of multiple muscle groups and placing high demands on the joints, particularly during sport-specific activities (2). Upper-limb (UL) asymmetry has received increasing attention in sports science because differences between limbs may influence physical performance and sport-specific skills (3, 4). While some degree of asymmetry is considered a normal characteristic of human movement, greater asymmetries have been associated with reduced performance and the adoption of compensatory movement strategies, particularly in athletes with underlying neuromuscular impairments (5, 6).

Within this context, a significant proportion of wheelchair athletes present UL asymmetries, frequently associated with imbalances in muscular strength and/or restricted joint range of motion due to different impairments (7, 8). However, the origin of these asymmetries may reflect distinct, albeit overlapping, mechanisms: sport-specific technical demands (e.g., throwing or striking); impairment-related motor limitations (e.g., muscle weakness, impaired trunk control or spasticity); and the repetitive mechanical loading associated with wheelchair propulsion (7–10). Consequently, the asymmetry profile observed in a given athlete may reflect the interaction between impairment characteristics and sport participation. During para-sport participation, athletes are exposed to a wide range of movement demands, including both symmetrical actions (e.g., wheelchair propulsion and weightlifting) and asymmetrical tasks (e.g., throwing or catching a ball). Each of these movement patterns imposes specific mechanical and neuromuscular demands that may be further influenced by impairment-related constraints on performance (11).

Across these activities, the UL and trunk are constantly exposed to internal and external forces, which can lead to significant physical overload and, in some cases, asymmetry in the muscle activation or joint range of motion (ROM) among athletes (7, 8). Several studies emphasize the importance of muscular balance, as asymmetries may alter muscle behavior during different phases of wheelchair propulsion, thereby generating increased mechanical stress on the shoulder (9, 12). These effects may be particularly relevant in athletes with spinal cord injury, who rely heavily on shoulder musculature for mobility and sport participation (13). However, the magnitude and characteristics of asymmetry may differ substantially according to both the athlete's impairment profile and the technical requirements of the sport practiced. Despite the growing interest in asymmetry among para-athletes, comparative evidence across wheelchair sports remains limited. Understanding the degree of asymmetry within specific para-sports disciplines may support the optimization of training protocols and wheelchair-specific movement patterns (14). Therefore, this study aimed to compare UL asymmetry levels among wheelchair athletes across different para-sports disciplines and to categorize them according to their level of asymmetry through an exploratory analysis. We hypothesised that UL asymmetry levels would vary according to para-sport discipline, allowing sports to be classified into lower- and higher-asymmetry profiles based on their specific functional and technical demands.

## 2 Methods

### 2.1 Participants

A convenience sample of 30 para-athletes with physical disabilities (men = 27 and women = 3; age = 31.43 ± 10.14 years; mass = 75.67 ± 16.32 kg; height = 170.65 ± 10.92 cm) was recruited from different para-sport competitive disciplines to voluntarily participate in the study. Inclusion criteria required participants to be wheelchair users actively competing at the international level as members of the Chilean national Paralympic team during the 2024 season. Sports represented included powerlifting, wheelchair rugby, table tennis, wheelchair basketball, boccia, and wheelchair handball. Exclusion criteria comprised UL injury within the previous six months, ongoing post-surgical recovery, acute UL pain ≥5/10 on the Visual Analog Scale on the day of testing, or cognitive impairment that prevented adherence to instructions. Before enrolment, all participants received a detailed explanation of the study's purpose, assessment procedures, potential risks, and expected benefits. After receiving this information, they provided written informed consent. Athletes and coaches were also invited to provide feedback on the evaluation procedures. The study was approved by the Ethics Committee of the Universidad de los Andes (code: K1230) and conducted in accordance with the Declaration of Helsinki, following the STROBE guidelines for observational studies.

### 2.2 Procedures

This exploratory cross-sectional study evaluated UL muscle strength and ROM in wheelchair para-athletes representing six sport disciplines, with the aim of characterizing asymmetry profiles across disciplines. All assessments were conducted during a single testing session at the same location to ensure consistency across participants. Prior to testing, participants received a detailed explanation of the protocol, including demonstrations of each movement, and were allowed to perform familiarization trials to minimize potential learning effects. A standardized warm-up consisting of ten minutes of light wheelchair propulsion and dynamic UL mobilization exercises was performed to prepare athletes for maximal exertion. Testing was scheduled outside of competitive events and training sessions to avoid acute fatigue.

### 2.3 Strength and ROM assessment

UL strength and ROM were assessed using the ActivForce2 device (Activbody, San Diego, CA, USA), a portable dynamometer/inclinometer paired with an iOS-compatible mobile application, which has been previously validated and applied in research settings (15). This device provided real-time measurements of muscle strength (N) and ROM (°) across the different testing procedures. Assessments were conducted with athletes seated in their sport-specific wheelchairs, with the wheels locked and with trunk and legs stabilized to ensure proper execution of the required movements. The strength evaluation protocol comprised six standardized isometric glenohumeral tasks and one elbow flexion task, performed bilaterally (dominant and non-dominant sides). Each trial was executed at maximal voluntary isometric effort for three seconds against resistance applied at the distal forearm, accompanied by standardized verbal encouragement (“push as hard as possible, push, push…”), with one-minute rest intervals between repetitions (15). All measurements were performed by a single experienced physiotherapist following a standardized protocol. Prior to data collection, the device was calibrated using the Activforce 2 application on an iPhone 13.

**Figure 1:**
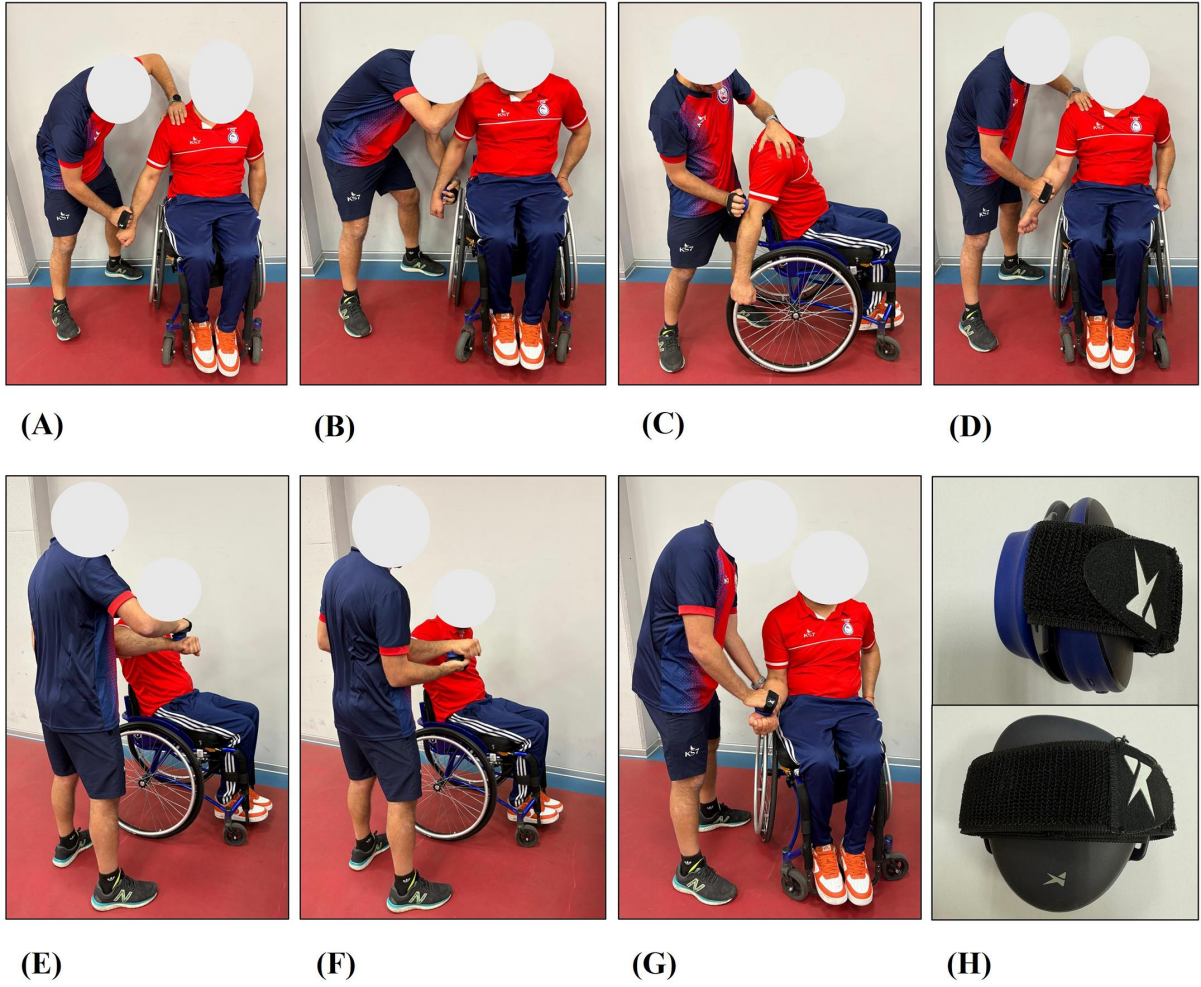
Sequential images illustrating the movements assessed using the ActivForce2 **(H)** device. **(A)** Shoulder abduction; **(B)** shoulder adduction; **(C)** shoulder extension; **(D)** shoulder flexion; **(E)** shoulder external rotation; **(F)** shoulder internal rotation; **(G)** elbow flexion.

Shoulder abduction was assessed at 45°, adduction at 10° flexion with full elbow extension, and flexion/extension from a neutral adducted position. Internal and external rotations were measured with the shoulder and elbow both at 90° abduction/flexion, and the forearm pronated. Except for the rotational tasks, all movements were executed with the elbow fully extended, ensuring consistent leverage and resistance application across conditions (Figure 1). Elbow flexion was assessed with the elbow flexed to 90°, using the wheelchair as support. ROM was assessed bilaterally using the ActivForce2 device positioned at the hand, measuring abduction/adduction in the frontal plane, flexion/extension in the sagittal plane, internal/external rotation with the shoulder and elbow at 90°, and elbow flexion from full extension to maximal range. In all cases, athletes actively moved through their maximal pain-free range, and the device recorded end-range joint angles in degrees, along with symmetry percentage between the right and left sides. For each task, three trials were recorded, and the highest value was considered the maximum strength.

As an exploratory method, asymmetry (%) was calculated using the formula: [(Dominant − Non-dominant)/Dominant]  × 100, where positive values indicate greater strength in the dominant limb (16). The dominant side was determined by the athlete's self-report and corroborated by the limb used to manipulate the sport-specific implement. For classification, the absolute asymmetry was used: values ≥15% were deemed asymmetric, following early research (16). Each sport discipline was classified as symmetric (<15%) or asymmetric (≥15%) based on the group-average UL asymmetry index across seven muscle strength assessments.

### 2.4 Statistical analysis

Descriptive statistics (i.e., mean ± standard deviation) were used to summarise the anthropometric characteristics of each sport (powerlifting, wheelchair rugby, table tennis, wheelchair basketball, boccia, and wheelchair handball). The Shapiro–Wilk test was applied to assess the normality of the strength and ROM asymmetry indices across seven movements: shoulder abduction, adduction, flexion, extension, internal rotation, external rotation, and elbow flexion. To explore potential differences between symmetric and asymmetric profiles, comparisons were conducted using either the Student's *t*-test (shoulder abduction, flexion, internal rotation, and external rotation strength) or the Mann–Whitney *U*-test (shoulder adduction, extension, and flexion strength, and all ROM variables), selected according to data distribution normality. For each variable, results were reported as mean ± standard deviation and median. Furthermore, Cohen's d was considered to inform the effect size between the two groups (0.20 = small, 0.50 = medium, 0.80 = large, 1.30 = very large) (17). All analyses were conducted using GraphPad Prism (version 10, GraphPad Software, San Diego, CA, USA), with statistical significance set at *p* < 0.05.

## 3 Results

The sample comprised 30 para-athletes: 3 powerlifting, 7 wheelchair rugby, 6 table tennis, 9 wheelchair basketball, 3 boccia, and 2 wheelchair handball (27 men and 3 women). All participants had eligible impairments for competition in their respective wheelchair sports, requiring the use of a wheelchair both in sport and in daily life. Participant health medical conditions included spinal cord injury, cerebral palsy, Duchenne muscular dystrophy, Charcot-Marie-Tooth, hip dysplasia, myelomeningocele, and lower-limb amputation. Spinal cord injury was the most prevalent in the sample (*n* = 14), with cervical-level lesions being the most common (Table 1).

**Table 1:** Demographic characteristics by sport, including health condition, age, weight, and height. Data are presented as mean and standard deviation (SD).

Sports	Health condition	Sport Class	Age (years)	Weight (kg)		Height (cm)		
			mean	(SD)	Mean	(SD)	mean	(SD)
Table tennis (*n* = 6)	Spinal cord injury, Duchenne muscular dystrophy	*ITTF*: C1, C1, C2, C2, C4, C4	29.17	(6.01)	74.00	(11.21)	174.33	(10.13)
Boccia (*n* = 3)	Cerebral palsy, spinal cord injury, muscular dystrophy	*BISFed*: BC2, BC2, BC4	29.33	(11.02)	70.00	(37.16)	159.00	(11.00)
Wheelchair rugby (*n* = 7)	Charcot-Marie-Tooth, spinal cord injury	*IWRF*: 0.5, 0.5, 1.0, 1.0, 2.0, 2.0, 2.0	37.00	(3.83)	73.71	(18.24)	173.00	(15.17)
Wheelchair handball (*n* = 2)	Spinal cord injury	*IHF*: 2.0, 3.0	55.50	(3.54)	73.50	(2.12)	172.00	(2.83)
Wheelchair basketball (*n* = 9)	Cerebral palsy, spinal cord injury, amputation, myelomeningocele, hip dysplasia	*IWBF*: 1.0,1.5, 2.0, 2.5, 3.0, 3.5, 4.0, 4.0, 4.5	26.13	(4.26)	81.13	(10.52)	171.00	(10.38)
Powerlifting (*n* = 3)	Cerebral palsy, spinal cord injury	*WPPO*: sport class	25.67	(7.23)	73.67	(12.58)	172.67	(6.43)

The international classification systems differ across the six wheelchair Para-sports. In Para table tennis, wheelchair classes range from 1 to 5, with class 1 indicating greater functional limitation and class 5 greater sitting, trunk, arm, and hand function (ITTF: International Table Tennis Federation). Boccia includes classes BC1–BC4, differentiated by locomotor function, ball-delivery capacity, and permitted assistance (BISFed: Boccia International Sports Federation). Wheelchair rugby classes range from 0.5 to 3.5, with 0.5 representing the greatest impairment-related activity limitation (IWRF: International Wheelchair Rugby Federation). Wheelchair handball comprises four classes, from class 1, indicating the lowest motor capacity, to class 4, indicating the highest eligible motor capacity (IHF: International Handball Federation). In wheelchair basketball, classes range from 1.0 to 4.5 and are determined by functional capacity and volume of action, particularly trunk stability (IWBF: International Wheelchair Basketball Federation). In Para powerlifting, all eligible athletes compete within a single sport class and are separated by gender and bodyweight category (WPPO: World Para Powerlifting Organization).

As an exploratory method, sports were classified as symmetric or asymmetric based on a 15% threshold strength. According to this criterion, wheelchair handball, wheelchair basketball, and powerlifting were categorised as symmetric, with mean total asymmetry values of 10.00 ± 7.44%, 12.17 ± 3.28%, and 10.47 ± 4.92%, respectively. In contrast, table tennis, boccia, and wheelchair rugby were classified as asymmetric, with mean total asymmetry values of 17.54 ± 6.72%, 30.27 ± 10.34%, and 26 ± 11.26%, respectively (Table 2).

**Table 2:** Mean shoulder and elbow strength by sport symmetry classification (*n* = 30).

Sports	Shoulder Abduction (%)	Shoulder Adduction (%)	Shoulder Flexion (%)	Shoulder Extension (%)	Shoulder Internal Rotation (%)	Shoulder External Rotation (%)	Elbow Flexion (%)	Seven movements assessed (%)	Classification
	mean (SD)	mean (SD)	mean (SD)	mean (SD)	mean (SD)	mean (SD)	mean (SD)	Total mean (SD)	
Table tennis (*n* = 6)	22.60 (13.90)	11.00 (8.70)	6.00 (2.90)	22.30 (17.10)	19.50 (10.50)	24.10 (13.20)	17.30 (12.10)	**17.54** (**6.72)**	asymmetric
Boccia (*n* = 3)	32.90 (22.40)	50.20 (31.20)	35.30 (10.10)	25.60 (24.30)	25.80 (11.03)	20.40 (24.10)	21.70 (15.40)	**30.27** (**10.34)**	asymmetric
Wheelchair rugby (*n* = 7)	18.00 (11.70)	20.70 (18.80)	29.90 (10.80)	46.10 (64.50)	30.70 (17.40)	27.90 (20.30)	11.00 (7.90)	**26.33** (**11.26)**	asymmetric
Wheelchair handball (*n* = 2)	6.30 (6.30)	7.30 (7.20)	4.70 (3.20)	2.80 (3.70)	16.10 (13.10)	23.90 (1.00)	8.90 (4.40)	**10.00** (**7.44)**	symmetric
Wheelchair basketball (*n* = 9)	13.40 (7.00)	10.40 (7.00)	6.10 (4.10)	12.30 (7.60)	11.90 (9.40)	15.80 (12.70)	15.30 (20.40)	**12.17** (**3.28)**	symmetric
Powerlifting (*n* = 3)	16.40 (3.10)	7.30 (4.20)	6.80 (5.80)	14.80 (9.30)	7.50 (10.60)	4.80 (3.20)	15.70 (14.70)	**10.47** (**4.92)**	symmetric

The asymmetric sports showed significantly greater strength than the symmetric sports in shoulder abduction (22.51% vs. 13.02%; *p* = 0.03, *d* = 0.84), flexion (21.95% vs. 6.06%; *p* = 0.00, *d* = 1.42), extension (20.34% vs. 9.58%; *p* = 0.04, *d* = 0.68), and internal rotation (25.62% vs. 11.55%; *p* = 0.00, *d* = 1.08). Similarly, ROM was significantly greater in the asymmetric sports for shoulder abduction (11.20% vs. 4.17%; *p* = 0.00, *d* = 0.95), adduction (20.80% vs. 11.98%; *p* = 0.03, *d* = 0.80), and flexion (7.62% vs. 3.27%; *p* = 0.02, *d* = 0.66), with both patterns predominantly observed in table tennis, boccia, and wheelchair rugby (Table 3).

**Table 3:** Mean strength and range of motion (ROM) percentages in asymmetric (*n* = 16) and symmetric (*n* = 14) sport groups.

	**Shoulder Abduction**		**Shoulder Adduction**		**Shoulder Flexion**		**Shoulder Extension**		**Shoulder Internal Rotation**		**Shoulder External Rotation**		**Elbow Flexion**	
	mean (SD)	median	mean (SD)	median	mean (SD)	median	mean (SD)	median	mean (SD)	median	mean (SD)	median	mean (SD)	median
Strength in asymmetric sports (%)	22.51 (14.51)	25.78	22.89 (22.32)	13.98	21.95 (15.20)	22.99	33.32 (44.46)	20.34	25.62 (15.62)	21.84	25.09 (17.58)	24.33	16.55 (12.07)	14.85
Strength in symmetric sports (%)	13.02 (6.64)	13.42	9.26 (6.22)	9.66	6.06 (4.06)	5.20	11.47 (8.00)	9.58	11.55 (9.59)	9.60	14.63 (11.71)	12.76	14.48 (17.21)	8.81
Effect size (Cohen's d)	0.84		0.83		1.42		0.68		1.08		0.70		0.13	
*p*—value	*0.03		0.05		*0.00		*0.04		*0.00		0.06		0.37	
ROM in asymmetric sports (%)	14.11 (13.08)	11.20	29.25 (16.57)	20.80	13.86 (17.25)	7.62	24.01 (24.05)	18.28	9.24 (7.26)	8.28	43.38 (29.23)	43.08	9.56 (12.96)	3.42
ROM in symmetric sports (%)	4.91 (3.97)	4.17	17.76 (11.60)	11.98	5.38 (4.87)	3.27	21.46 (25.34)	14.86	11.38 (8.19)	9.96	28.03 (21.63)	23.75	8.56 (5.31)	9.56
Effect size (Cohen's *d*)	0.95		0.80		0.66		0.10		0.27		0.56		0.10	
*p*—value	*0.00		*0.**03**		*0.02		0.42		0.55		0.06		0.37	

* *p* > 0.05 for comparisons between the asymmetric and symmetric sport groups for both strength and range of motion.

SD, Standard deviation.

## 4 Discussion

This study compared UL asymmetry across athletes of different wheelchair para-sports and identified differences between disciplines classified as symmetric and asymmetric. While wheelchair basketball, wheelchair handball, and powerlifting showed relatively balanced UL profiles, athletes from table tennis, boccia, and wheelchair rugby exhibited marked strength asymmetries, with values exceeding the 15% exploratory threshold. Moreover, significant intergroup differences were observed in key strength measures (i.e., shoulder abduction, flexion, extension, and internal rotation) and in selected ROM outcomes (i.e., shoulder abduction, adduction, and flexion), with consistently higher values recorded in the asymmetric sports, suggesting that sport-specific demands and impairment levels could shape UL functional adaptation over time. However, these findings should be interpreted with caution, since the relatively small number of athletes representing certain sport disciplines, combined with the functional heterogeneity of para-athletes (i.e., diverse health conditions), limits the generalizability of the observed asymmetry patterns. Therefore, future investigations should prioritize increasing sample sizes across all disciplines to allow more robust comparisons.

Regarding our variables, a distinctive pattern was shown, which reflects differences in both muscular strength and ROM, which could be consistent with the biomechanical demands inherent to each sport discipline. In this regard, Soltau et al. described that wheelchair propulsion in able-bodied participants without impairments and pain can be assumed as symmetrical, although bigger asymmetries can be expected in those with neurological health conditions (18). For instance, wheelchair basketball and handball are intermittent sports requiring frequent changes of direction, sprinting, accelerations, decelerations, and technical skills, all of which may promote more balanced use of the UL (19, 20). Similarly, powerlifting involves bilateral, explosive movements to lift maximal loads. Although impaired athletes have shown higher asymmetries compared to their able-bodied peers, kinetic analyses suggest relatively symmetrical bilateral muscle activation (7, 21). The present findings may therefore be related to the lower impairment levels of these athletes, including better trunk control, UL function, and overall mobility, which favor more symmetric profiles (22, 23). Indeed, the interplay between impairment severity and movement symmetry could become particularly evident when contrasting these sports that consistently exceeded the asymmetry threshold. However, these variables were not assessed in the present study.

Conversely, those sports classified as more asymmetrical based on the specified threshold included only one team sport with intermittent demands (i.e., wheelchair rugby) (24), para-table tennis that uses exclusively one arm (25), and boccia, which focuses on athletes with high support needs (26). These findings may suggest an increased risk of shoulder overload during wheelchair propulsion in certain disciplines. However, this interpretation remains preliminary, as the relevant variables were not assessed in the present study. In wheelchair rugby, athletes often present impairments affecting all four limbs, and previous research has linked impaired trunk strength to greater asymmetry (24, 27). Interestingly, longer sprint distances have been associated with reduced peak power asymmetries, suggesting possible compensatory motor adjustments to overcome functional limitations (28). Indeed, Brassart et al. reported that rugby players showed a subtle reduction in starting asymmetry compared to basketball athletes (29), potentially due to these adaptations.

Asymmetries are common in table tennis, particularly in the frontal and transverse planes, characterized by intense one-sided trunk muscle work to meet sports demands, which increase these specific asymmetries (30). In the case of para-table tennis, the high technical demands characterized by rapid trunk rotations, repeated unilateral strokes, and constrained postural control inherently promote greater asymmetry compared with other wheelchair sports that involve more bilateral propulsion patterns (30, 31). These sport-specific technical demands may partly explain the greater asymmetry observed in strength and ROM. In wheelchair para table tennis, the repetitive use of the racket arm during high-speed forehand and backhand strokes, together with restricted trunk motion and the stabilizing and propulsive demands placed on the contralateral limb, may produce side-specific neuromuscular and musculoskeletal adaptations (25, 32). Consequently, these asymmetries likely reflect the combined influence of technical specialization, wheelchair use, trunk function, and impairment characteristics.

In contrast, boccia involves athletes with high support needs and severe impairments, including quadriplegic cerebral palsy. Boccia is inherently characterized by asymmetrical movement patterns, as its precision-based throwing actions rely predominantly on one UL and are further constrained by limited trunk stability in athletes with severe impairments (33). Given that Boccia players often present significant neurological impairments, the combination of reduced trunk control and altered coordination likely contributes to the higher asymmetry observed in this population (33, 34). Therefore, the asymmetry detected in our study may be interpreted as a reflection of both the sport's technical demands, unilateral, repetitive throws, and the functional limitations of boccia athletes, which together shape performance outcomes. Consistently, previous research has highlighted that wheelchair athletes, due to neuromuscular features, muscular imbalances, restricted ROM, and equipment-related adaptations, present factors that increase both asymmetry and injury risk (35). Taken together, these findings reinforce the notion that asymmetries are highly complex and context dependent, being not only sport-specific, as demonstrated in able-bodied populations, but also deeply shaped by impairment-specific characteristics that interact with the technical demands of each discipline (36).

## 5 Limitations

This study presents some limitations that must be acknowledged. First, the sample size was relatively small (*n* = 30) and drawn exclusively from the Chilean national Paralympic teams, which may limit the generalizability of the findings to broader populations of wheelchair athletes. The heterogeneous distribution of impairments (e.g., spinal cord injury, cerebral palsy, muscular dystrophy) and uneven representation across sports disciplines (e.g., only two participants in wheelchair handball) may also have influenced the variability of asymmetry outcomes. Similarly, pooling disciplines into symmetric and asymmetric sports may have limited the identification of sport-specific characteristics and introduced some degree of variability within each group. For this reason, the findings should be interpreted cautiously.

The use of a portable dynamometer and inclinometer provided valid measures of muscle strength and range of motion, but these assessments were limited to isometric tasks and did not include dynamic or sport-specific movement patterns that may provide further ecological validity. Finally, the absence of a control group of able-bodied wheelchair users limits comparisons between impairment-related and sport-related asymmetries. Future studies should aim to include larger and more diverse samples across countries and competition levels to increase external validity. Longitudinal designs are warranted to examine the progression of asymmetries over time, their relationship with training exposure, and potential associations with injury incidence.

## 6 Conclusion

This exploratory study identified discipline-specific patterns of UL asymmetry among wheelchair para-athletes. Athletes competing in table tennis, boccia, and wheelchair rugby, which comprised the asymmetric sport group, exhibited the greatest asymmetries, particularly in shoulder internal rotation and abduction strength and in shoulder adduction and abduction ROM. These differences likely reflect the influence of sport-specific technical demands and neuromuscular adaptations associated with each athlete's impairment profile. However, given the exploratory nature of this study and the absence of potentially relevant variables, including athlete-specific performance metrics and functional kinematics during play, the findings should be interpreted with caution and considered preliminary.

Moreover, these findings may have practical implications for coaches, clinicians, and Paralympic classification systems. Discipline-specific asymmetry benchmarks could support individualized training programs, contribute to injury-prevention strategies, and provide complementary information for sport-specific classification. Moreover, longitudinal monitoring to establish sport-specific asymmetry reference values could be essential for developing evidence-based guidelines for wheelchair para-athletes.

## Data Availability

The original contributions presented in the study are included in the article/Supplementary Material, further inquiries can be directed to the corresponding author.
